# Aging increases the systemic molecular degree of inflammatory perturbation in patients with tuberculosis

**DOI:** 10.1038/s41598-020-68255-0

**Published:** 2020-07-09

**Authors:** Deivide Oliveira-de-Souza, Caian L. Vinhaes, María B. Arriaga, Nathella Pavan Kumar, Artur T. L. Queiroz, Kiyoshi F. Fukutani, Subash Babu, Bruno B. Andrade

**Affiliations:** 1 Fundação Oswaldo Cruz, Instituto Gonçalo Moniz, Salvador, 40296-710 Brazil; 2 Multinational Organization Network Sponsoring Translational and Epidemiological Research (MONSTER) Initiative, Salvador, 41810-710 Brazil; 3 Curso de Medicina, Faculdade de Tecnologia e Ciências (UniFTC), Salvador, 40290-150 Brazil; 4 International Center for Excellence in Research, National Institutes of Health- National Institute for Research in Tuberculosis, Chennai, 600031 India; 5 Laboratory of Parasitic Diseases, NIAID, NIH, Bethesda, 20892 USA; 6 Escola Bahiana de Medicina e Saúde Pública (EBMSP), Salvador, 40290-000 Brazil; 7 Laureate Universities, Universidade Salvador (UNIFACS), Salvador, 41720-200 Brazil; 8 Wellcome Centre for Infectious Diseases Research in Africa (CIDRI-Africa), Institute of Infectious Disease and Molecular Medicine, University of Cape Town, Cape Town, 7925 South Africa

**Keywords:** Immunology, Cytokines, Infection, Infectious diseases, Inflammation

## Abstract

Tuberculosis (TB) is a chronic infection that can affect individuals of all ages. The description of determinants of immunopathogenesis in TB is of tremendous interest due to the perspective of finding a reliable host-directed therapy to reduce disease burden. The association between specific biomarker profiles related to inflammation and the diverse clinical disease presentations in TB has been extensively studied in adults. However, relatively scarce data on profiling the inflammatory responses in pediatric TB are available. Here, we employed the molecular degree of perturbation (MDP) score adapted to plasma biomarkers in two distinct databanks from studies that examined either adults or children presenting with pulmonary or extrapulmonary disease. We used multidimensional statistical analyses to characterize the impact of age on the overall changes in the systemic inflammation profiles in subpopulation of TB patients. Our findings indicate that TB results in significant increases in molecular perturbation, with the highest values being detected in adult patients. Furthermore, there were unique differences in the biomarker perturbation patterns and the overall degree of inflammation according to disease site and age. Importantly, the molecular degree of perturbation was not influenced by sex. Our results revealed that aging is an important determinant of the differences in quality and magnitude of systemic inflammatory perturbation in distinct clinical forms of TB.

## Introduction

Tuberculosis (TB) remains the leading cause of mortality worldwide due to a single agent^1^. *Mycobacterium tuberculosis* (Mtb) is widely disseminated geographically and infects individuals of all ages, causing a wide spectrum of clinical manifestations associated with the host immunological status^2^.

The majority of the studies exploring immunopathogenesis in TB is restricted to the adult population. On the other hand, TB pathophysiology remains poorly understood in children, especially in those under 5 years-old^3–5^. An important challenge in pediatric TB is the increased frequency of extrapulmonary presentations^6^, which can be paucibacillary and thus associated with challenges in microbiologic test confirmation, resulting in delayed therapy implementation.

Inflammatory biomarkers in TB have been extensively studied and have gained prominence due to the potential use as host-based blood tests^7^. Understanding the complexity of the inflammatory milieu during TB infection in children is the key to expand the field and drive development of new tools to aid the clinical management and contribute to non-sputum-based point-of-care tests.

Here, we used an adaptation of the molecular degree of perturbation (MDP), as previously published by us^8,9^, to assess the nuances of systemic inflammation associated with TB in pediatric and adult patient populations in an exploratory study. We found that the degree of inflammatory imbalance is associated with aging, suggesting that TB patients develop augmented capacity of promoting systemic inflammation with increasing age. Given that TB clinical presentation is a result of immunopathology, our results reinforce the idea that the distinct inflammatory profile in blood underlies the differences observed in TB disease presentation between adults and children.

## Materials and methods

### Study design and participants

Active TB cases were recruited at the Government Stanley Medical Hospital, at TB clinics supported by the National Institute for Research in Tuberculosis and Childs Trust Hospital in Chennai, India. Detailed information on diagnosis of PTB in adults have been described previously^8,10^, whereas the procedures used for diagnosis of PTB and EPTB in children have been already reported^11,12^. Briefly, the diagnosis of PTB in adults and children was based on sputum smear and culture positivity. EPTB in adults was diagnosed on the basis of AFB staining and/or culture positivity of fine-needle aspiration biopsies of lymph nodes or pleural effusions in adult. The diagnosis of EPTB in children was made on clinical symptoms, physical examination and biopsies according to the site of clinical manifestation, such as fine-needle aspiration for the cases of TB lymphadenitis or cerebrospinal fluid analysis for TB meningitis as described in^12^. At the time of enrollment, all active TB cases had no record of prior TB disease or anti-TB treatment (ATT). The healthy control adults were asymptomatic with normal chest X-rays, negative TST (indurations < 5 mm in diameter) and QuantiFERON TB Gold-in-Tube enzyme-linked immunosorbent assay (Cellestis), as well as negative sputum smear or culture results as described in^8,10^. Pediatric participants included in the heathy control group were asymptomatic who went to the hospital for routine vaccinations and tested negative in the QuantiFERON TB assay. All participants were BCG vaccinated (had BCG scar) and were HIV negative. Plasma samples were collected from a total of 152 adults and 54 children. Among adults, 97 were diagnosis of PTB, 35 of EPTB and 20 were healthy controls. Within children, 14 had PTB, 22 had EPTB and 18 were healthy controls. Adults with EPTB had TB lymphadenitis (n = 24) or pleural TB (n = 11) whereas children presented with spinal TB (n = 14), TB lymphadenitis (n = 6), and abdominal TB (n = 2), which included peritonitis or tuberculomas. Considering previously published data and study power calculations on cytokine measurements^12^, we found that a sample size of n = 10 per group would be result in a study power of 90% with alpha error of 5% to find at least 1.5-fold-difference (which was an assumption) between levels of the overall MDP scores between HC and PTB or EPTB groups. To potentially find the same results in the adult population, a larger sample (n = 20) was required. Thus, our study was overpowered to find such differences. All participants were recruited in Chennai, India, as part of a large TB natural history study.

### Immunoassays

We evaluated two databanks containing plasma concentrations of a panel of 17 cytokines, tissue remodeling mediators and matrix metalloproteinases to examine molecular degree of inflammatory perturbation using different immunoassays. The was no pre-selection of biomarkers. Only biomarkers which concentrations were recorded in databank from both children and adults were used for the analysis. In the original study, biomarkers were measured in EDTA-treated plasma samples. Biomarkers included were cytokines, acute-phase proteins and tissue remodeling proteins. Bio-Plex luminex assay system (R&D Systems) was employed to measure the cytokines analyzed. The list of cytokines included interleukin (IL)-1β, IL-10, IL-12p70, IL-17, interferon (IFN)-γ and tumor necrosis factor α (TNF-α). Moreover, IFN-α and IFN-β levels were quantified using the VeriKine serum ELISA kit (PBL Interferon Source). Plasma levels of vascular endothelial growth factor (VEGF) was measured using the Milliplex map kit system by Merck Millipore. Concentration of extracellular matrix metalloproteinases (MMPs)-1, 8 and 9, and of the tissue inhibitors of metalloproteinases (TIMPs)-1, 2, 3 and 4 were measured using Luminex technology (R&D Systems), according to the manufacturer's protocols. Plasmatic Hemoxygenase 1 (HO-1) was measured by ELISA (Assay Designs).

### Data analysis

Categorical data were presented as proportions and continuous data as medians and interquartile ranges (IQR). The Fisher’s exact test (2 × 2) or the Pearson’s chi-square test was used to compare categorical variables between study groups. Continuous variables were compared using the Mann–Whitney *U* test (between 2 groups) or the Kruskal–Wallis test with Dunn’s multiple comparisons ad hoc test. P-values were adjusted for multiple measurements using the Holm–Bonferroni’s method. Hierarchical cluster analyses (Ward’s method) of z-score normalized data were employed to depict the overall expression profile of indicated biomarkers in the study groups. Dendrograms represent Euclidean distance.

Profiles of correlations between biomarkers in different clinical groups were examined using network analysis of the Spearman correlation matrices (with 100 × bootstrap). In indicated analyses, only correlations with significant adjusted P-values (established cut-off was P-value < 0.003) were included in the network visualization. In such analyses, markers that exhibited similar correlation profiles were clustered based on a modularity^13^, which infers sub-networks inside the of the correlation network profiles, and depicted using Fruchterman Reingold (force-directed graph drawing).

An analytical algorithm using sparse canonical correlation analysis (CCA) was employed to assess whether data on correlation between combinations of circulating biomarkers could discriminate between subgroups of patients. The CCA model was chosen because many variables were studied. This model is able to perform dimensionality reduction for two co-dependent data sets (MDP biomarker profile and baseline characteristics profile, which were age and sex) simultaneously so that the discrimination of the clinical endpoints represents a combination of variables that are maximally correlated. Thus, trends of correlations between parameters in different clinical groups rather than their respective distribution within each group are the key components driving the discrimination outcome. In our CCA model, simplified and adapted from previously reported investigations of biomarkers for TB diagnosis^8,14–17^ and of inflammatory pathways associated with pathogenesis of other infectious diseases^18^. In the biomarker profile dataset, we included values of all the inflammatory marker. The diagnostic class prediction values obtained were calculated using receiver operator characteristics curve analysis. Probability of being molecularly perturbed according to increases in age was calculated using Kaplan–Meyer curves.

### Adaptation of the molecular degree of perturbation to examine plasma concentrations of biomarkers

The plasma measurements of both datasets were normalized equally with a log2 transformation and the batch effect within the different study datasets was corrected using *Comba*t algorithm from SVA package^15^. The *ComBat* algorithm is a widely used method for adjusting batch effects in microarray and RNA-Seq data associated with technical variance effects. The molecular inflammatory perturbation is based molecular degree of perturbation (MDP) method used in the present study is an adaptation of the MDH described by Pankla et al.^19^. In the present study, instead of using gene expression values as in Prada-Medina et al.^20^, we inputted plasma concentrations of a defined set of biomarkers which were described in previously published studies from our group that investigated TB pathogenesis^14,21^. Thus, herein, the average plasma concentration levels and standard deviation of a baseline reference group (healthy uninfected controls) were calculated for each biomarker. The MDP score of an individual biomarker was defined by the differences in concentration levels from the average of the biomarker in reference group divided by the reference standard deviation. Essentially, the MDP score represents the differences by number of standard deviations from the healthy control group. The formulas used to calculate MDP in the present study are shown below:

$$Molecular \,degree\, of \,perturbation\,=\,\frac{{\text{x}}_{\text{i}}- {\overline{\text{x}}}_{(\text{r}\text{e}\text{f}\text{e}\text{r}\text{e}\text{n}\text{c}\text{e})}}{{\upsigma }_{(\text{r}\text{e}\text{f}\text{e}\text{r}\text{e}\text{n}\text{c}\text{e})}}$$

$$\upsigma =\frac{{\sum }_{\text{i}=1}^{\text{n}}{({\text{x}}_{\text{i}}-\overline{\text{x}})}^{2}}{\text{n}-1}$$

n = Number of data points, x_i_ = Each of the value of data, $\overline{{\text{x}}}$ = Mean of the data points, σ = Standard deviation.

In the present study, we applied the MDP scoring system using data on 17 biomarkers measured from two distinct groups of patients, adults and children with active TB and healthy uninfected controls. The MDP transformation was used as an approach to normalize data cross experiments resulting in datasets with markers distributed in a similar scale.

### Ethics statement

All clinical investigations were conducted according to the principles expressed in the Declaration of Helsinki. Written informed consent was obtained from all participants or their legally responsible guardians. The study was approved by the Institutional Review Board of the National Institute for Research in Tuberculosis, Chennai, India (NIRT; protocol numbers NCT01154959 and NCT00342017).

## Results

### Characteristics of study participants

Baseline characteristics of the adult and pediatric participants have been described elsewhere^12^. Adults with pulmonary TB (PTB) and uninfected healthy controls were more frequently males than those with extrapulmonary TB (EPTB) (65.9% vs. 85% vs. 45.7%, respectively; P = 0.006). In addition, adults with PTB were on average older than healthy controls (median (IQR) in years: 38 (28–47) vs. 28.5 (26–35), respectively; P = 0.005) but had median age similar to the group of EPTB (Supplementary Table S1). In the pediatric study, patients with PTB were similar to those presenting with EPTB or HC with regard age (median (IQR) in years: 6.5 (1.7–12.5) vs. 7 (3.7–13) vs. 10 (3–12), respectively; P = 0.684) and sex (P = 0.393), with and overall high frequency of male individuals (42.8% vs. 59.1% vs. 66.7%) (Supplementary Table S2). We next compared the adults and children and found that there were no significant differences in sex distribution in comparisons between the HC, the PTB as well as the EPTB subgroups (Supplementary Table S3).

### Increases in molecular degree perturbation of plasma cytokines and tissue remodeling mediators in adult and children with active tuberculosis

Plasma levels of 17 cytokines and tissue remodeling mediators were compared between PTB and EPTB and uninfected healthy controls (HC) in adult and children from India, separately (concentration values are described in Supplementary Tables S4, S5). In adults, compared to HC, the PTB or EPTB groups exhibited higher levels of most parameters, except for IFN-β, TIMP-2, IL-12p70 and TIMP-4 which were not statistically different (Supplementary Table S4). In the pediatric population, individuals with active TB (PTB or EPTB) exhibited on average higher levels of fewer markers (HO-1, MMP-1, MMP-8, TIMP-1 and TIMP-3) than controls (Supplementary Table S5). These results suggested that TB in adults may lead to more significant changes in the concentration levels of plasma biomarkers than what we observed in children with this condition.

**Figure 1:**
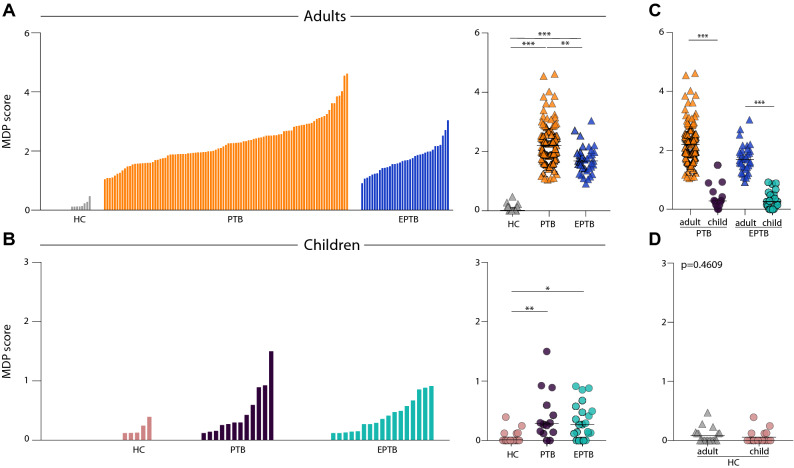
Adult and children with active tuberculosis exhibit substantial molecular degree of perturbation. (**A**,**B**) Left panels: Histograms show the single sample molecular degree of perturbation (MDP) score values relative to the healthy control group between adult and child (*PTB* pulmonary TB, *EPTB* extrapulmonary TB, *HC* healthy controls). MDP values were calculated as described in Methods. The Kruskal–Wallis test with Dunn’s multiple comparisons was used to compare MDP values between each clinical group. Right panels: Scatter plots of the summary data for each group are shown. MDP score values were compared between PTB or EPTB patients (**C**) or healthy controls (**D**) from Adult and Child. Lines in the scatter plots represent median values. Data were compared using the Mann–Whitney *U* test. *P < 0.05; **P < 0.01; ***P < 0.0001.

The data described above were originated from two different cohorts^10,12^. In order to directly compare the groups from the distinct studies, we calculated the overall MDP score values according to our previous publication^8^ and found that active TB was associated with a substantial increase in MDP scores compared to HC in both adults (PTB P < 0.0001, EPTB P < 0.0001; Fig. 1A) and children (PTB P < 0.001, EPTB P = 0.002; Fig. 1B). Adult patients with PTB exhibited higher MDP values than those with EPTB (P = 0.0007; Fig. 1A), whereas MDP values in PTB and EPTB were indistinguishable in children (P > 0.999; Fig. 1B). Interestingly, adult patients with either PTB or EPTB exhibited higher MDP values than those from the pediatric population (adults PTB vs. children PTB: P < 0.0001; adults EPTB vs. children EPTB: P < 0.001; Fig. 1C) suggesting that the impact of Mtb on changes in the systemic molecular degree of perturbation is likely influenced by age. Reinforcing this idea, we found no differences in MDP values between adults and children from the HC group (P = 0.46; Fig. 1D).

### Plasma markers driving the overall molecular degree of perturbation in tuberculosis are distinct between adults and children

**Figure 2:**
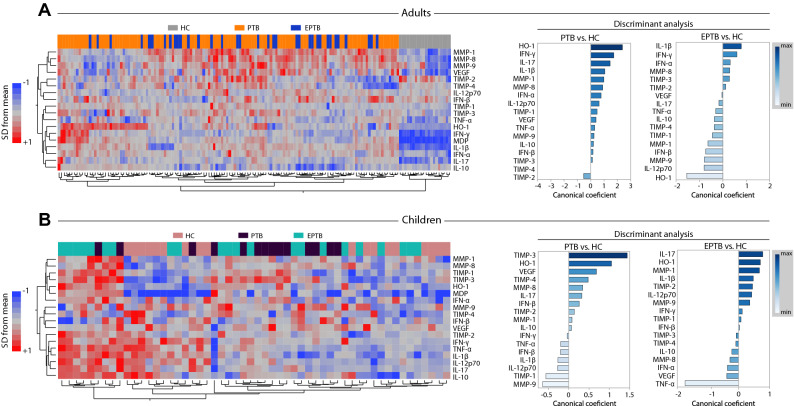
Plasma biomarkers driving the overall molecular degree of perturbation in pulmonary tuberculosis are distinct between adults and children. (**A**,**B**) Left panels: Unsupervised two-way hierarchical cluster analyses (Wards method with 100 × bootstrap) using the MDP values (z-score normalized) for each individual markers measured in plasma from patients from both groups were employed to test if simultaneous assessment of such markers could group PTB or EPTB separately from healthy individuals. Dendrograms represent Euclidean distance. Right panels: A discriminant analysis model based on canonical correlation analyses was used to identify the markers which are driving the discrimination between the study groups. Number of patients per group: Adult HC: n = 20, Adult PTB: n = 97, Adult EPTB: n = 35, Child HC: n = 18, Child PTB: n = 14, Child EPTB: n = 22.

We examined the MDP expression values for each individual plasma cytokine and tissue remodeling mediators. An unsupervised hierarchical clustering was used to test whether perturbation values of all the markers combined were associated with specific changes that could characterize active TB (PTB or EPTB) and HC in adults and children. We found that the overall expression profile was very distinct between adults with active tuberculosis and HC (Fig. 2A, left panel). Intriguingly, children showed heterogenous profile expression of MDP values of all the 17 markers between active TB and HC (Fig. 2B, left panel), without a clear separation in the cluster analysis. These findings indicate that the overall expression profile of the MDP values can be used to distinguish active TB from controls in adults but not in children. They also suggest that although the overall perturbation was substantially higher in children with active TB, perturbation of individual markers was not sufficient to reliably segregate those from the healthy control group. In addition, PTB could not be grouped separately from EPTB in both adults and children (Fig. 2A,B, left panels), indicating that active TB drives specific changes in MDP independent on disease site. Univariate analyses comparing the MDP values for each marker between HC and PTB or EPTB groups are shown in Supplementary Figs. S1 and S2.

Furthermore, we employed a sparse canonical correlation analysis using MDP values^8^ to test whether the profile of correlations between the plasma markers, rather than their individual concentration values, could be used to distinguish PTB or EPTB and HC in both adults and children. Curiously, the markers with the strongest contributions for discrimination in adults with PTB vs. HC were HO-1, IFN-γ, IL-17, IL-1β and TIMP-2 whereas in children such markers were TIMP-3, HO-1, VEGF, TIMP-4 and MMP-9 (Fig. 2A,B right panels). The canonical model also specified that the markers which contributed the most for the discrimination between EPTB and HC in adults were IL-1β, IFN-γ, IFN-α, MMP-8 and HO-1, whereas in children were IL-17, HO-1, MMP-1, IL-1β and TNF-α (Fig. 2A,B right panels). Of note, the MDP values of 3 out of 5 markers which were relevant to identify PTB in adults (HO-1, IFN-γ and IL-1β) were also part of TB signature observed in adults with EPTB. Moreover, in children, only 1 marker which was relevant to identify PTB (HO-1) was also relevant to highlight EPTB. These findings indicate that in the context of Mtb infection, the plasma markers likely to be differentially perturbed according to disease site and age are distinct, except for HO-1.

### Network correlation profiles of molecular degree of perturbation in active TB are distinct between adults and children

**Figure 3:**
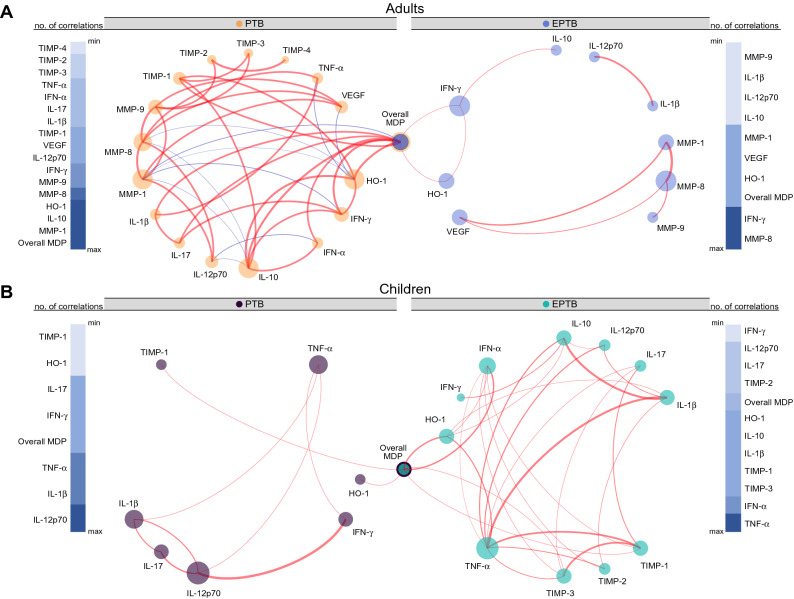
Network analysis of the MDP matrices in the study groups. (**A**,**B**) Spearman correlation matrices of the biomarker expression levels in each study group were built and Circos plots were used to illustrate the correlation networks. Each circle represents a different plasma parameter. The size of each circle is proportional to the number of significant correlations. P-values were adjusted for multiple measurements using Holm–Bonferroni’s method and the connecting lines represent statistically significant correlations (P < 0.003). Red connecting lines represent positive correlations while blue lines infer negative correlations. Color intensity is proportional to the strength of correlation (rho value). Node analysis was used to illustrate the number of significant correlations per marker. Markers were grouped according to the number of connections from minimum to maximum numbers detected.

To understand the nuances between molecular degree of perturbation of individual markers and their direct effect on overall MDP values, we employed network analysis based on Spearman correlation matrices, as previously described^8^. Using this approach, we found that the presence of pulmonary infection in adults was associated a greater number of correlations when compared with those that developed EPTB (Fig. 3A). The group of adults with PTB was marked by several positive correlations, highlighting that the degree of perturbation in HO-1, IFN-α, IFN-γ, IL-10, IL-17, IL-1β and TIMP-1 markers was directly associated with the overall MDP score values (Fig. 3A, left panel). In addition, perturbation of MMP-1 levels was inversely correlated with the overall MDP values in this clinical group. Furthermore, the top nodes exhibiting the highest number of significant correlations in the network of adults with PTB were the overall MDP score followed by MMP-1, IL-10, HO-1 and MMP-8. Importantly, the correlation profile found in adults with EPTB was distinct from PTB (Fig. 3A). Indeed, only the degree of perturbation of HO-1 and IFN-γ were directly associated with the overall MDP values in the EPTB group. Node analysis of the EPTB network demonstrated that MMP-8, IFN-γ, overall MDP and HO-1 were the most highly connected parameters (Fig. 3A, right panel).

When the network analysis was extended to the pediatric population, we observed a decreased number of statistically significant correlations in pulmonary infection when compared with extrapulmonary TB (Fig. 3B). Interestingly, we found that Mtb infection was associated with marked absence of negative correlations in children. Node analysis of the PTB network indicated that IL-12p70, IL-1β and TNF-α were the most highly connected markers (Fig. 3B, left panel). Curiously, the overall MDP values, which were highly connected in the networks from adults, were statistically correlated only with perturbation of HO-1 and TIMP-1. Children with extrapulmonary TB exhibited TNF-α, followed by IFN-α, TIMP-3 and TIMP-1 as the most relevant nodes (Fig. 3B, right panel). The degree of perturbation of HO-1, IFN-α, TIMP-1 and TIMP-3 was directly associated with the overall MDP values in children with EPTB. Furthermore, in children, there was a lack of correlations between the MDP values of markers described to be important in TB pathogenesis such as IFN-γ, IL-1β and TNF-α in the networks. These findings argue that age influences the correlation between specific molecular markers that drive the overall molecular perturbation in active TB.

### Age directly influences the overall inflammatory perturbation profile in active tuberculosis independent of sex

**Figure 4:**
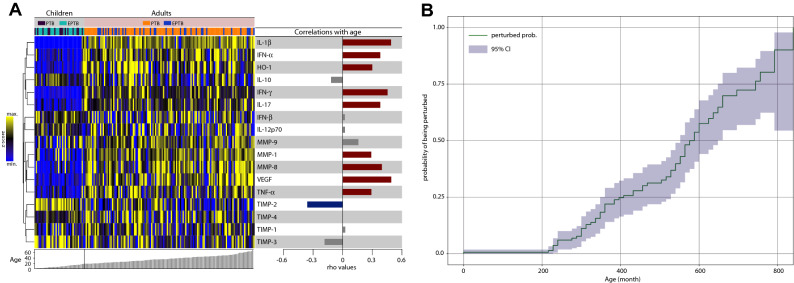
Associations between molecular degree inflammatory perturbation and age in TB patients. (**A**) Molecular degree of perturbation was assessed in samples from adults and children with tuberculosis. Data were z-score normalized. A hierarchical cluster analysis was employed to group the biomarkers based on their overall expression profile in the study population. Dendrograms represent Euclidean distance. Each individual was grouped based on age. The right panel shows Spearman correlation coefficient values of relationships between the indicated parameters and age. (**B**) The Kaplan–Meier curve shows the probability of being molecularly perturbed according to the age. Spearman correlation rank was compared used Steger Method.

The results described above suggested that age was associated with the molecular degree of inflammatory perturbation. To directly test this hypothesis, we grouped each individual based on age and performed an exploratory investigation using unsupervised hierarchical cluster analysis with z-score normalized values of the MDP calculated for each marker. We found that PTB did not exhibit a distinct biomarker MDP profile compared to EPTB independent on age (Fig. 4A, left panel). Of note, MDP values detected for many markers were relatively lower in children independent on the TB clinical presentation, except for TIMP-1, TIMP-2, TIMP-3 and TIMP-4, which tended to be higher in those who were younger (Fig. 4A, left panel). We next tested direct correlations between age and the individual MDP values of each marker. Spearman correlation analysis revealed that the degree of perturbation of IFN-γ, IL-1β, TNF-α, IFN-α, HO-1, IL-17, MMP-1, MMP-8 and VEGF was directly correlated whereas TIMP-2 values were inversely correlated with age (Fig. 4A, right panel).

**Figure 5:**
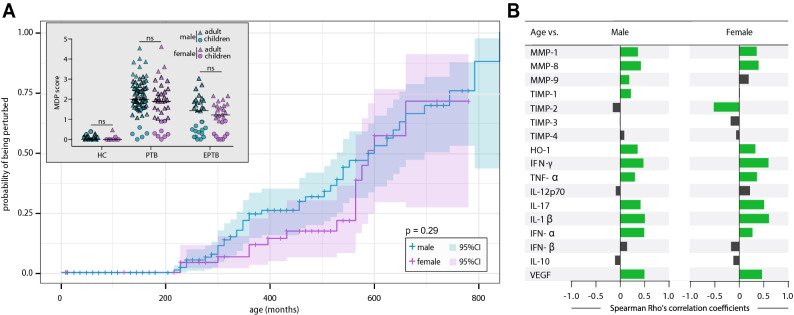
Molecular degree of perturbation is independent of biological sex in patients with active tuberculosis. (**A**) Perturbed probability according to the age. Spearman correlation rank was compared used Steger Method. (**B**) Spearman correlation analysis was used to test association between age and MDP values of each biomarker in either male or female TB patients. Bars represent the Spearman rank (rho) values. Colored bars indicate statistically significant correlation (P < 0.05) after adjustment for multiple measurement.

To determine association between age and probability of being molecularly perturbed (see Methods for definition) in the entire population, we used a model adapted from the Kaplan–Meier survival curve (Fig. 4B). This approach revealed that increase in age was directly associated with higher probability of overall molecular perturbation. Indeed, the overall MDP score values were positive correlated with age in all the clinical groups evaluated (Supplementary Fig. S3). We next analyzed the association between age and MDP values of each marker in our clinical groups, in adults or in children. We found that there were differences in regard to the relationship between aging and variation of MDP values of individual biomarkers in the distinct clinical groups in adults (Supplementary Fig. S4A) as well as in children (Supplementary Fig. S4B). However, the vast majority of these differences were not statistically significant (adjusted P-values ≥ 0.05). Finally, we examined the influence of sex on the association between age and inflammatory perturbation. Overall MDP values were not different between male and female individuals stratified in the distinct clinical groups (Fig. 5A, insert). Furthermore, using the Kaplan–Meier survival curve test, we found that, in general, there was no different in the curves of female and male participants (P = 0.29, Fig. 5A). In fact, it was possible to notice that females tended to be less perturbed between 300 and 600 months of age, however with a considerable variation represented by large confidence intervals (denoting the low precision of the assessment) which overlapped with those from the curve of male participants (Fig. 5A). Furthermore, Spearman correlation analysis demonstrated that MDP values of most of markers were positively correlated with age in both male and female participants (Fig. 5B). TIMP-2 was the only marker that displayed a negative correlation in females. These findings suggested that sex does not substantially influence the higher probability of inflammatory perturbation with aging in TB.

## Discussion

Mechanisms of disease pathogenesis in TB have been extensively studied over the years, but despite that, the immunopathology of this infection in pediatric populations remains poorly understood. Importantly, Mtb infection is one of the main causes of childhood morbidity and mortality worldwide^11,22,23^, and the field needs elucidation of the determinants of immunopathogenesis. In the present study, we used an adaption of molecular degree of perturbation^8,9^ to estimate the level and quality of systemic inflammation in patients with active TB (PTB and EPTB) according to age. Our findings indicate that there are important discrepancies in the MDP values between adults and children with active TB, with adults exhibiting higher values, whereas the molecular perturbation was similar among individuals from the healthy control groups independent of age. Thus, while the systemic inflammatory profile is similar between adults and children without TB, it becomes very distinct in patients with active disease. Although we have not directly tested potential influence of maturation of immune system in the results, it is possible that the capacity to promote significant systemic inflammation in the context of TB may be affected by this process.

Mycobacterial infection is known to cause profound stimulation of both innate and adaptive immune response, in vitro and in vivo models^24,25^. As a consequence, differential activation of leukocytes, such as monocytes^26,27^ and lymphocytes^28,29^, has been used to characterize the disease and even to serve as potential diagnostic biomarkers for PTB and EPTB, as recently reported by our group^7^. Whether differential activation of immune cells in tissue or in peripheral blood upon Mtb infection are the underlying factor dictating the systemic degree of perturbation described here is an assumption to be tested in future studies. Our exploratory analyses characterized the systemic inflammatory response and indicated that Mtb infection (pulmonary and extrapulmonary) was associated with overall increases in the MDP values in both adults and children. However, in either PTB or EPTB groups, adult patients exhibited augmented MDP values compared to pediatric patients. This finding reinforces the idea that in the context of TB, adults are more prone towards presenting with a higher degree of inflammation than children. We expanded these analyses to show the degree of perturbation of each individual biomarker and found that the in adults, individuals with active TB exhibit a very distinct profile compared to those without. Nevertheless, in the pediatric population, there was no clear combined biomarker profile that could distinguish TB from controls. Hence, there are specific changes in the biomarker MDP profile that are age dependent. Of note, using discriminant analyses based on a canonical correlation model, we identified that the top markers responsible for the discrimination between the clinical groups differed between adults and children. In adults, HO-1, IFN-γ and IL-1β, markers which have been associated with TB pathogenesis^30–32^ were the top markers that contributed to discrimination between the disease groups (both PTB and EPTB) and controls^14,30,33^, whereas in children, TIMP-3, IL-17 and HO-1 were the markers that most contributed for discrimination between active TB patients and uninfected controls. Of note, one important finding of this analytical approach was that the molecular perturbation of HO-1 could discriminate pulmonary and extrapulmonary TB from healthy controls in both adult and children populations. HO-1 is important marker of oxidative stress response, highly expressed in the lungs^10,34^, with a critical role in cytoprotection^35^. These observations made us hypothesize HO-1 may be important in TB pathogenesis regardless of age. Additional studies in more diverse populations are warranted to test this idea.

The inflammatory process results from an intricate relationship between factors from the host and pathogen, and can be evaluated using network analysis^9,36,37^. Using this approach, we showed important differences in the correlation profiles between MDP values from each individual biomarker and the overall MDP values in adults and children with active TB. Pulmonary infection with Mtb in adults led to a coordinated inflammatory burst, which was read by detection of many statistically significant correlations between individual markers and overall MDP values. On the converse, in children presenting with PTB the augmented inflammation seemed less coordinated, meaning increases in molecular perturbation of a given marker were not followed by simultaneous increases of other markers or the overall MDP values. This suggests that pediatric PTB patients may have a decreased ability to mount a coordinated immune response. It is reasonable to hypothesize that such uncoupling of inflammatory activity may be a consequence of immune immaturity that leads to dissemination of bacilli in children, as previously demonstrated^6^. Curiously, in children with EPTB, the networks were more complex, indicating a higher number of connections. Such phenomenon could be consequence of extrapulmonary tissue damage, and maybe argue that despite the lower capacity to mount and sustain a coordinated response in pulmonary infection, EPTB in children is marked by a more balanced interplay between innate and adaptive immune response^24^. Importantly, IFN-α, a relevant cytokine in the context of TB pathogenesis, was one of those most highly connected markers in children with EPTB, being also connected to overall MDP expression values. This finding suggests that IFN-α may be a parameter influencing or being influenced by the pathological inflammation which characterizes EPTB. Moreover, in adults with EPTB, the complexity of the inflammatory network was reduced, in agreement with the previous published evidence of probably less organized and an unfettered immune activation in adults with EPTB^9^. The determinants of the differences in correlations between concentrations of mediators of inflammation between distinct clinical forms of TB in adults and children are still unknown.

An interesting result reported here was the association between increases in age and augmentation of the overall molecular degree of perturbation in both PTB and EPTB patients. Growing old has been associated with increased basal inflammatory state that alters susceptibility to many diseases, including TB^38^. Our results demonstrated that increase in age leads to rise in probability of a TB patient becoming more perturbed, independent on disease site (PTB or EPTB), mainly after 200 months. Importantly, we described that degree of perturbation of many individual plasma markers correlated with age, reinforcing the idea that the capacity to induce systemic inflammation is proportional to age. In pediatric patients, immune activation has several peculiarities compared to that in adults, which result from a continuous evolution of the immune system from infancy to old age^39^. Fetal and neonatal immune adaptations facilitate intrauterine survival and provide early postnatal protection against extracellular pathogens, but they leave infants susceptible to intracellular pathogens such as viruses that are acquired perinatally^40^. Contrasting immune activation profiles between children and adults have been reported in the context of infection with Mtb^41^, *Helicobacter pylori*^42^, HIV^43^, SARS-Cov-2^44^, and by several other pathogens. In these settings, the immune response described in children are frequently more modest, less intense than what is observed in adults. It is known that failure of alveolar macrophages to contain Mtb and recruit additional mononuclear cells to the site of infection helps to explain the more fulminant course of TB in early life^41^. Moreover, as far as in the current covid-19 pandemics, it has been reported that infected adults can develop different outcomes, from asymptomatic, mild, moderate to severe disease, and death. Children, on the other hand, can also be infected by SARS-CoV-2, but most cases with laboratory-confirmed infection are mild and severe disease is rare^44,45^. Our results lead us to speculate that children infected with Mtb do not substantially activate systemic inflammation as well as in adults. Intriguingly, our findings suggest that the probability of being perturbed in the context of TB is dramatically increased after 200 months of age. It is possible that, in highly endemic regions such as South India, there is an accumulation of exposures to Mtb as well as to other infectious agents during the childhood. Such gradual exposure could result in increased capacity to induce an augmented degree of systemic inflammation, which is proportional to the inflammatory perturbation assessed here through the MDP score. Additional studies are necessary to really define if this is an intrinsic characteristic of pediatric patients of is conditioned to certain diseases. Future investigations are also required to directly test whether the response of immune cells from individuals with TB at different age ranges are reflected by the differential probability of being molecularly perturbed. Furthermore, our analysis showed that biological sex did not influence the probability of increase in MDP values, arguing that the potential effect of sex-related hormones on systemic inflammation may be superposed by the effect of aging in TB patients.

In India, BCG is part of the national vaccination schedule and offered to all newborns. In our study, both pediatric and adult population exhibited the BCG scar and thus were vaccinated. Therefore, it is difficult to discriminate the effect of BCG vaccination on the concentration values of plasma cytokines in such setting. The details on long-lasting effects of BCG on systemic levels of cytokines are not completely understood. Two possible immunological mechanisms have been suggested to describe the nonspecific effects of BCG vaccination. The first mechanism is heterologous immunity, in which cross-protection is mediated by heterologous T-cell memory responses^46^. The studies investigating this hypothesis fail to describe meaningful changes in plasma levels of cytokines; they rather show increased capacity of cells to respond in vitro upon stimulation^46^. A second mechanism of protection has been recently proposed in the form of epigenetic reprogramming of immune cells, a phenomenon conferring nonspecific immune memory to innate immune responses and termed ‘trained immunity’^47^. Indeed, BCG vaccination in healthy volunteers has been reported to induce epigenetic reprogramming of monocytes, leading to increased cytokine production in response to nonrelated pathogens for up to 3 months after vaccination^48^. In addition, such vaccine is capable of inducing boosted heterologous Th1/Th17 responses, which could persist in up to 1 year after vaccination^49^. Although to our knowledge there is no formal demonstration of the long-lasting effect of BCG on plasma levels of mediators of inflammation, it is possible that BCG vaccination had a potential impact on the circulating levels of cytokines in the pediatric participants. It is known that BCG triggers important effects on innate responses, mainly in macrophages and natural-killer cells^50,51^, leading to increases in IL-1β and TNF-α, aside from IFN-γ^52^. Our findings demonstrated slightly higher expression of IL-1β in healthy controls, higher TNF-α in children presenting with EPTB and increased levels of IFN-γ in children with PTB, but such differences were not statistically significant. The MDP score values of these three important cytokines were all significantly higher in adults with EPTB or PTB compared to their matched disease subgroups in children whereas the values between the HC groups were indistinguishable. Future studies specifically testing the effects of BCG on systemic immune responses and whether such effects drive changes in the molecular degree of perturbation are warranted.

Our study has limitations. We were unable to test association between MDP and bacillary loads due to lack of data from the pediatric population. It is possible that higher MDP values detected in adults may be a consequence of the increased mycobacterial infection loads. In addition, the sample size of the group of children was relatively small, although the it was sufficient to result in a substantial study power. Regardless, the extensive exploratory analyses performed revealed unique relationships between age and the systemic degree of inflammation. The results presented here shed light into the impact of aging on the systemic immune activation during TB.

## Supplementary information

Supplementary Information

## Data Availability

The datasets generated during and/or analyzed during the current study are available from the corresponding author on reasonable request.
