# Supplementary material for: Aging increases the systemic molecular degree of inflammatory perturbation in patients with tuberculosis
Source: Sci Rep. 2020 Jul 9;10:11358. doi: 10.1038/s41598-020-68255-0 (PMC7347549; doi:10.1038/s41598-020-68255-0)
Supplement: Supplementary file 1 — Supplementary Information [file 41598_2020_68255_MOESM1_ESM.pdf]

# Aging increases the systemic molecular degree of inflammatory perturbation in patients with tuberculosis

Deivide Oliveira-de-Souza, Caian L. Vinhaes, María B. Arriaga, Nathella Pavan Kumar, Artur T. L. Queiroz, Kiyoshi F. Fukutani, Subash Babu, Bruno B. Andrade

## Supplemental Tables and Figures

### Table S1. Characteristics of adult participants

| Characteristic | Healthy controls | PTB | EPTB | P-value |
|---|---|---|---|---|
| N | 20 | 97 | 35 | |
| Age – y | 28.5 (26-35) | 38 (28-47) | 35 (25-45) | 0.0452 |
| Male – no. (%) | 17 (85) | 64 (65.9) | 16 (45.7) | 0.0062 |

Data represent medians and interquartile ranges (age) and frequencies (male sex). The Kruskal-Wallis test was used to compare distributions of age while the Pearson's chi-square test was used to compare frequencies.

**Table S2. Characteristics of pediatric participants**

| Characteristic | Healthy controls | PTB | EPTB | P-value |
|---|---|---|---|---|
| N | 18 | 14 | 22 | |
| Age – y | 10 (3-12) | 6.5 (1.7-12.5) | 7 (3.7-13) | 0.6841 |
| Male – no. (%) | 12 (66.7) | 6 (42.8) | 13 (59.1) | 0.3928 |

Data represent medians and interquartile ranges (age) and frequencies (male sex). The Kruskal-Wallis test was used to compare distributions of age while the Pearson's chi-square test was used to compare frequencies.

**Table S3. Sex distribution in study population**

| Group | Sex | Adult | Children | P-value |
|---|---|---|---|---|
| HC | Male | 17 | 12 | 0.2603 |
| | Female | 3 | 6 | |
| PTB | Male | 64 | 6 | 0.1372 |
| | Female | 33 | 8 | |
| EPTB | Male | 16 | 13 | 0.4173 |
| | Female | 19 | 9 | |

Data represent number of individuals in each group/category. The Fisher's exact test was used to compare frequencies.

**Table S4. Distribution of the plasma concentrations of the mediators of inflammation in adult participants**

| Parameter | Unit | Healthy controls | PTB | EPTB | P-value |
|---|---|---|---|---|---|
| N | | 20 | 97 | 35 | |
| Heme Oxygenase-1 | ng/ml | 1.5 (1.3-1.6) | 5.6 (3.2-11.6) | 3.8 (2.3-6.5) | **<0.0001** |
| IFN-α | pg/mL | 3.2 (1-4.3) | 11.6 (7.9-17.3) | 8.3 (4.8-12.1) | **<0.0001** |
| IFN-β | pg/mL | 4.4 (2.2-5.5) | 2.9 (1.3-7.1) | 3.4 (1.5-5.9) | 0.8231 |
| IFN-γ | pg/mL | 20.6 (15-19.5) | 33.9 (29.8-35.3) | 29.8 (27.8-36.0) | **<0.0001** |
| IL-10 | pg/mL | 18.4 (16.43-20.85) | 21.0 (15.93-29.05) | 14.8 (10.3-21.0) | **0.0014** |
| IL-12p70 | pg/mL | 2.3 (1.6-3.3) | 2.2 (0.9-5.3) | 2.9 (1.5-6.9) | 0.2078 |
| IL-17 | pg/mL | 16.3 (43.1-493.1) | 27.1 (25.85-35.32) | 26.5 (23.2-30.0) | **<0.0001** |
| IL-1β | pg/mL | 2.8 (2.5-3.4) | 15.9 (12.9-20.1) | 13.1 (11.7-15.9) | **<0.0001** |
| MMP-1 | ng/mL | 0.07 (0.04-0.32) | 3.4 (1.9-5.3) | 2.9 (2.2-4.5) | **<0.0001** |
| MMP-8 | ng/mL | 13.9 (5.1-32.5) | 122.4 (54.1-202.3) | 88.4 (75.6-141.9) | **<0.0001** |
| MMP-9 | ng/mL | 133.7 (53.7-331.5) | 384.2 (255.0-510.8) | 271.6 (214.7-329.6) | **<0.0001** |
| TIMP-1 | ng/mL | 151.4 (136.0-166.2) | 180.0 (161.7-199.7) | 169.6 (153.3-186.5) | **<0.0001** |
| TIMP-2 | ng/mL | 232.9 (219.2-255.9) | 218.9 (199.4-238.7) | 223.0 (208.3-234.1) | 0.1001 |
| TIMP-3 | ng/mL | 12.4 (5.5-19.2) | 26.3 (15.9-43.9) | 19.8 (11.5-29.4) | **<0.0001** |
| TIMP-4 | ng/mL | 9.6 (8.4-10.8) | 10.3 (7.7-12.4) | 8.8 (7.5-10.9) | 0.2174 |
| TNF-α | pg/mL | 12.5 (10.2-17.5) | 21.3 (14.9-27.4) | 18.9 (15.5-23.9) | **0.0006** |
| VEGF | pg/mL | 26.0 (18.4-47.8) | 100.8 (57.9-165.5) | 71.5 (43.0-113.3) | **<0.0001** |

Data represent medians and interquartile ranges. The Kruskal-Wallis test was used to compare the distributions of the plasma mediators between the study groups. P-values in bold font are statistically significant.

**Table S5. Distribution of the plasma concentrations of the mediators of inflammation in pediatric participants**

| Parameter | Unit | Healthy controls | PTB | EPTB | P-value |
|---|---|---|---|---|---|
| N | | 18 | 14 | 22 | |
| Heme Oxygenase-1 | ng/ml | 0.5 (0.4-0.6) | 0.6 (0.5-1.0) | 0.9 (0.6-1.1) | **0.0005** |
| IFN-α | pg/mL | 209.7 (11.2-380.8) | 70.0 (10.9-87.9) | 135.6 (21.4-735.8) | 0.1903 |
| IFN-β | pg/mL | 347.9 (233.4-511.0) | 312 (261.1-466.0) | 286.8 (200.0-427.7) | 0.5615 |
| IFN-γ | pg/mL | 244.7 (111.4-812.5) | 377.4 (252.4-720.5) | 325.4 (178.3-892.6) | 0.4723 |
| IL-10 | pg/mL | 15.6 (0.4-80.4) | 11.3 (10.0-13.1) | 5.3 (0.5-97.0) | 0.8904 |
| IL-12p70 | pg/mL | 10.6 (5.8-69.4) | 16.8 (7.9-55.9) | 13.6 (7.1-48.8) | 0.8443 |
| IL-17 | pg/mL | 7.8 (5.8-25.6) | 9.6 (5.5-21.3) | 9.4 (7.1-26.7) | 0.7314 |
| IL-1β | pg/mL | 85.9 (13.9-174.2) | 28.6 (2.3-87.4) | 42.3 (8.2-478.9) | 0.5183 |
| MMP-1 | ng/mL | 0.3 (0.2-0.4) | 0.5 (0.4-0.6) | 0.5 (0.3-0.7) | **0.0028** |
| MMP-8 | ng/mL | 6.4 (4.0-9.0) | 8.2 (7.8-11.5) | 8.5 (6.1-11.3) | **0.0367** |
| MMP-9 | ng/mL | 17.8 (13.5-25.5) | 23.1 (17.6-25.9) | 21.1 (14.6-35.2) | 0.4514 |
| TIMP-1 | ng/mL | 2.3 (1.7-3.0) | 4.0 (2.6-5.3) | 3.4 (1.9-5.1) | **0.0263** |
| TIMP-2 | ng/mL | 1.4 (1.1-1.8) | 1.7 (1.4-2.1) | 1.6 (1.5-2.4) | 0.1981 |
| TIMP-3 | ng/mL | 0.2 (0.2-0.3) | 0.7 (0.5-0.8) | 0.4 (0.3-0.5) | **<0.0001** |
| TIMP-4 | ng/mL | 0.02 (0.02-0.03) | 0.02 (0.02-0.03) | 0.02 (0.01-0.04) | 0.8515 |
| TNF-α | pg/mL | 162.7 (37.1-1691.1) | 87.6 (53.4-203.4) | 197.1 (88.1-735.0) | 0.3645 |
| VEGF | pg/mL | 71.0 (56.0-136.6) | 111.9 (78.6-127.8) | 86.1 (56.0-105.8) | 0.3415 |

Data represent medians and interquartile ranges. The Kruskal-Wallis test was used to compare the distributions of the plasma mediators between the study groups. P-values in bold font are statistically significant.

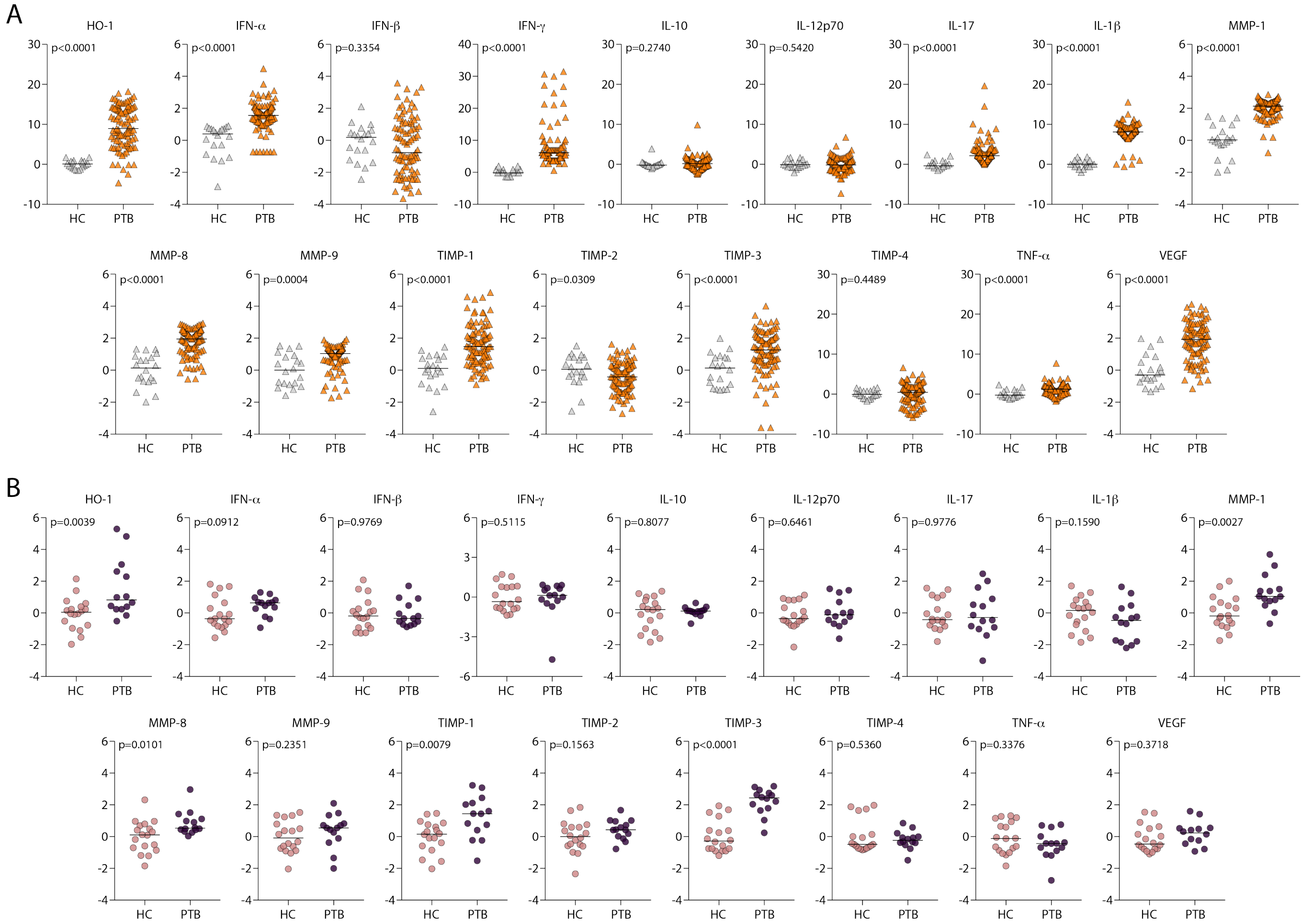


**Figure S1. Differences in the inflammatory perturbation of each plasma biomarker between active PTB and healthy control groups in either adults or children**

**(A,B)** Scatterplots of molecular degree of perturbation (MDP) of indicated biomarkers of TB patients stratified per the group (Adult HC, n=20 and PTB, n=97; Children HC, n=18 and PTB, n=14). MDP values are shown in Y-axis from all the plots. For this analysis, the MDP value of each given biomarker was calculated by the difference in concentration levels from the average of the biomarker detected in the reference group (HC) divided by the standard deviation from the HC group. Lines represent median values. Data were compared using the Mann–Whitney *U* test.

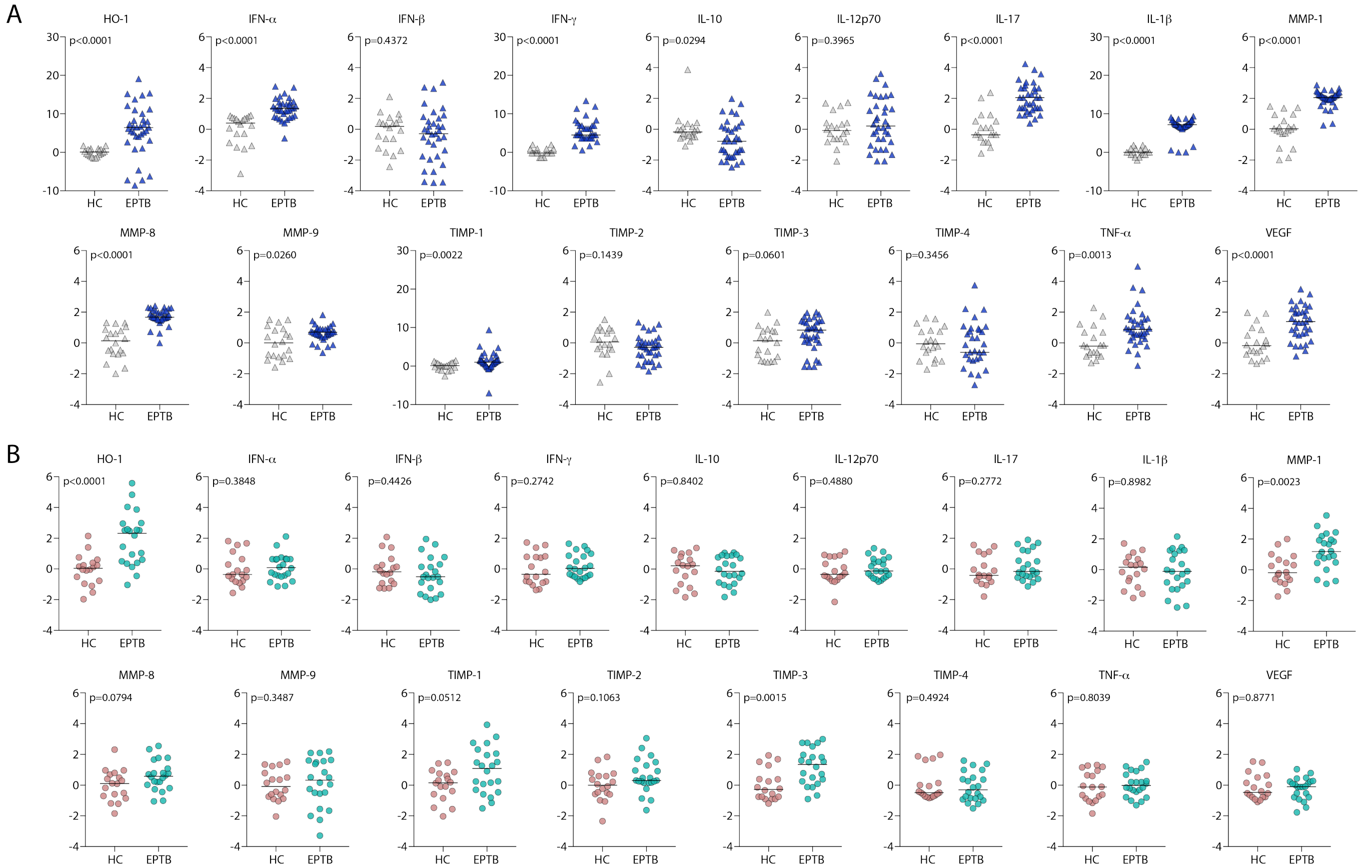


**Figure S2. Differences in the inflammatory perturbation between adult or children with extrapulmonary tuberculosis compared to healthy controls**

**(A,B)** Scatterplots of molecular degree of perturbation (MDP) of indicated biomarkers of TB patients stratified per the group (Adult HC, n=20 and EPTB, n=35; Children HC, n=18 and EPTB, n=22). MDP are shown in Y-axis from all the plots. For this analysis, the MDP value of each given biomarker was calculated by the difference in concentration levels from the average of the biomarker detected in the reference group (HC) divided by the standard deviation from the HC group. Lines represent median values. Data were compared using the Mann–Whitney *U* test.

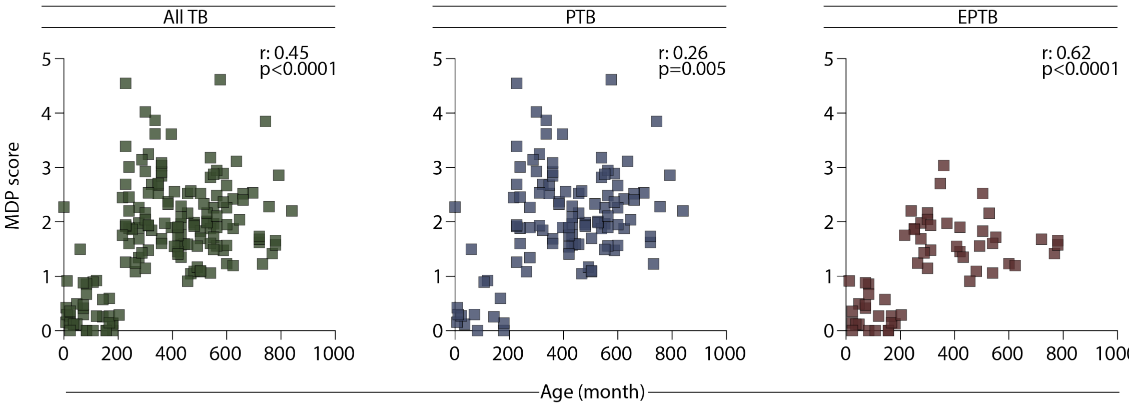


**Figure S3. Association between Age and overall MDP score values in patients with tuberculosis.**

Correlation between Age and MDP values for each biomarker was assessed using the Spearman rank test in the different study groups.

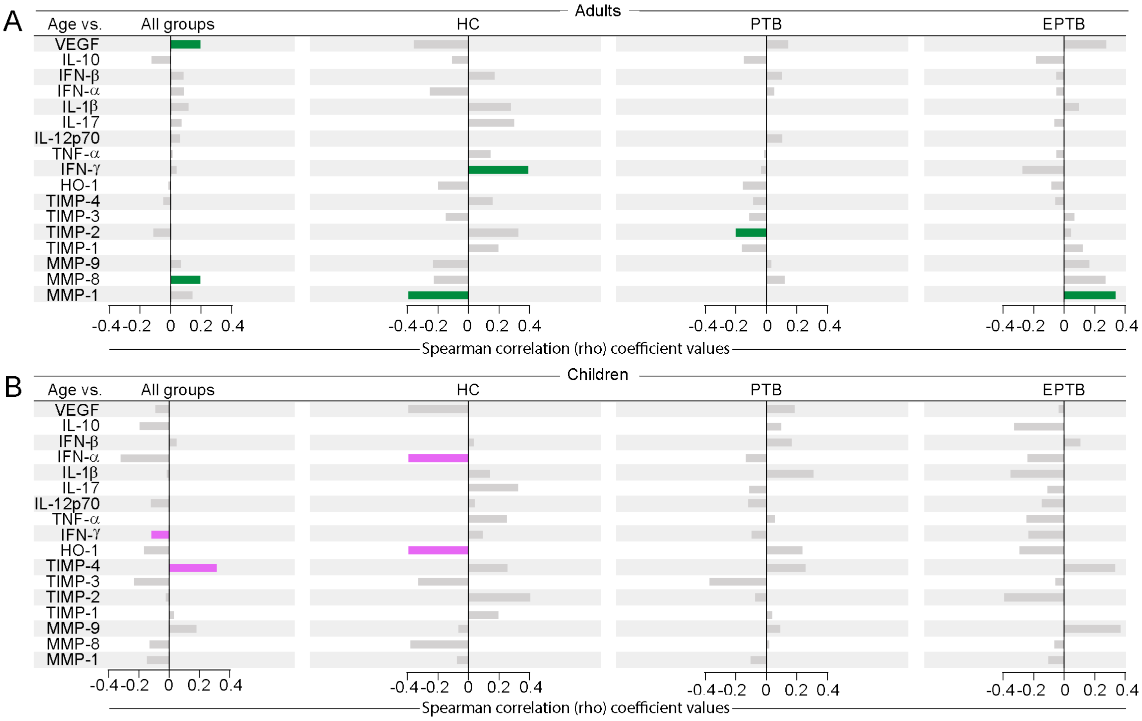


**Figure S4. Association between Age and MDP values for each biomarker per study group**

Correlation between Age and MDP values for each biomarker was assessed using the Spearman rank test in the different study groups, both in adults (A) and children (B). Bars represent the Spearman rank (rho) values. Colored bars indicate statistically significant correlation (P<0.05).
